# Skin bioprinting: a novel approach for creating artificial skin from synthetic and natural building blocks

**DOI:** 10.1007/s40204-018-0087-0

**Published:** 2018-05-12

**Authors:** Robin Augustine

**Affiliations:** Department of Mechanical and Industrial Engineering, College of Engineering, Qatar University, Doha, 2713 Qatar

**Keywords:** Bioprinting, Skin, Skin substitutes, Wound healing, Tissue engineering

## Abstract

Significant progress has been made over the past few decades in the development of in vitro-engineered substitutes that mimic human skin, either as grafts for the replacement of lost skin, or for the establishment of in vitro human skin models. Tissue engineering has been developing as a novel strategy by employing the recent advances in various fields such as polymer engineering, bioengineering, stem cell research and nanomedicine. Recently, an advancement of 3D printing technology referred as bioprinting was exploited to make cell loaded scaffolds to produce constructs which are more matching with the native tissue. Bioprinting facilitates the simultaneous and highly specific deposition of multiple types of skin cells and biomaterials, a process that is lacking in conventional skin tissue-engineering approaches. Bioprinted skin substitutes or equivalents containing dermal and epidermal components offer a promising approach in skin bioengineering. Various materials including synthetic and natural biopolymers and cells with or without signalling molecules like growth factors are being utilized to produce functional skin constructs. This technology emerging as a novel strategy to overcome the current bottle-necks in skin tissue engineering such as poor vascularization, absence of hair follicles and sweat glands in the construct.

## Introduction

Skin is the outermost protecting sheath of human body and is in direct contact with the external environment which makes it highly susceptible to injury. Skin defects or wounds are common which may result from trauma, skin diseases, burn or removal of skin during surgery (Coyer et al. 2015). Such circumstances require immediate therapeutic interventions to regain the structure and function of the skin and allow the usual mobility of the patient. Superficial wounds can lead to the bacterial invasion and related complications if not treated immediately (Horiuchi et al. 2010). Moreover, even minor deformities bring psychological distress on the affected individuals, especially to children. The best option of skin tissue engineering is the use of autografts though it is limited by the amount and size of available grafts besides other factors such as creation of a secondary wound and other risks (Zöller et al. 2014). Other types of skin grafts such as allografts and possibly xenografts are associated with the risks of immune reactions and transmission of diseases besides some ethical and cultural issues (Nunery 2001). Wound dressing materials such as those based on polymers or their combinations with other substances have largely been developed but they are not living and hardly can be cellularized and replaced by native tissue (Abrigo et al. 2014). In this regard, tissue engineering holds great promises for improving the treatment of skin defects by providing solutions for the challenges such as lack of multi-layered native skin architecture and vascular networks in the constructs (Jank et al. 2017). This approach provides some solutions where besides biomaterial, living cells, biological or chemical signals are being used with the purpose of forming functional skin (Metcalfe and Ferguson 2007b). However, such conventional tissue engineering approaches suffer from inherent problems of non-homogeneous distribution of cells, failure to integrate and vascularize upon implantation with subsequent rejection of the implanted biomaterial along with formed skin (Verseijden et al. 2010). Although, some tissue engineered skin products are in market, many limiting factors such as vascularization through the skin substitute which remains a major and critical limiting factor in the clinical success (MacNeil 2007).

There are number of cell seeded skin substitutes which are available in the market, mostly based on prefabricated collagen scaffolds seeded with allogenic neonatal foreskin fibroblasts and keratinocytes (Shevchenko et al. 2010). Recent developments that employ cell friendly processing techniques which can incorporate cells in the process of manufacturing of the scaffold with the aim of providing injectable cell-laden gels is a highly promising approach in the clinical translation of skin substitutes (Zhao et al. 2016). Techniques such as electrospinning which does not employ cell damaging high temperatures have also been used to immobilize cells in situ (Yeo and Kim 2014). Nevertheless, the high electric potential applied during electrospinning may affect the cell growth. Moreover, limited availability of spinnable and cell friendly solvents and the polymers which are soluble in such solvents is a limiting factor (Augustine et al. 2016b).

Along with other promising technologies, 3D printing was recently brought as an important processing technique in the field of tissue engineering to replace the concept of scaffold-based tissue engineering with cell-laden constructs that have good control over cells and biomaterial organisation (Murphy and Atala 2014a). In principle, bioprinting operates in a way similar to the conventional 3D printing technology where the printing ink referred as “bioink” contains biomaterials and cells to produce tissues (Hospodiuk et al. 2017). Initial success stories in bioprinting were based on custom made or modified ink-jet printers to print endothelial and smooth muscle cells over Matrigel and collagen gel in 2D fashion (Xu et al. 2013). Bioprinting has greatly advanced in the last five years and become one of the most promising techniques in tissue engineering. Bioprinting technology aims to generate accurately controlled organized assemblies and resemble the complex architectures of native tissues. Perhaps the most obvious application of bioprinting is the generation of tissue engineered constructs with properties and architecture similar to that of native tissue (Kang et al. 2016). Using bioprinting technology, a variety of internal structures and pores ranging from a few to hundreds of micrometres in size can be created in hydrogels (Stanton et al. 2015). As a result, it is possible to generate different layers of skin like stratum cornea, epidermis, papillary dermis, reticular dermis and the structures like vascular networks, sweat gland and hair follicles (Murphy and Atala 2014a). Tissue engineering strategies combined with bioprinting technology may greatly reduce the issues with graft failure, poor healing, limited vascularization, pathogen transfer, and immune rejection (Stanton et al. 2015).

In this review, we discuss and summarize the available information about skin bioprinting such as bioprinting methods, solidification strategies of the construct, effect of active agents in the scaffolds and loading of cells during bioprinting. We also summarize the outcomes, challenges and future prospects of skin bioprinting. A detailed discussion on the fabrication strategies for bioprinted tissue engineering scaffolds is beyond the scope of this particular review (Murphy and Atala 2014a). However, a brief introduction to bioprinting is provided. Importantly, the biomaterials used and their relevant properties related to skin tissue engineering are discussed. We also discuss some of the most commonly exploited natural and synthetic polymers, their blends and composites as bioinks. Finally, some of the most critical challenges and future approaches in skin bioprinting from bioengineering and clinical perspective are provided. We believe, we will witness a revolution in skin reconstruction through collaborative research and by putting combined expertise to the benefit of mankind by exploiting the advantages of bioprinting.

## A brief introduction to bioprinting: principles and technology

Bioprinting is an advanced manufacturing platform based on conventional 3D printing that enables the predefined deposition of biomaterials, living cells, and growth factors using computer-aided design (CAD) to fabricate custom designed tissue constructs by layer-by-layer printing process with a high degree of flexibility and repeatability (Murphy and Atala 2014a) (Ng et al. 2016a). Bioprinting technology has the potential to directly create graded macroscale architectures to better mimic the natural extra cellular matrix (ECM), thereby augmenting the attachment and proliferation of multiple types of cells concurrently. Bioprinting evolved from 3D printing required combined living cells to be seeded into the scaffolds in post-printing stage (Hockaday et al. 2012). At later stages, simultaneous printing of biomaterial matrix and cells was developed.

Post-printing seeding of cells may not result in the homogeneous cell distribution in the scaffold. This may also affect cell activity and results in tissue growth which making it difficult to have control over cells or tissue development. Thus, a homogeneous mixing of cells in a suitable hydrogel to form a bioink and subsequent printing avoids such bottlenecks (Markstedt et al. 2015). With the use of advanced 3D bioprinting approaches, it is possible to have precise control over the physico-mechanical and biological properties of the resulting scaffold. Controlling porosity and pore interconnectivity in skin substitutes, which is a big challenge besides other processing techniques, can be bypassed by bioprinting (Michael et al. 2013). Today, with the utilization of computer-driven bioprinters, precise deposition of cells and biomaterials, cell laden scaffolds with predetermined architectures can be achieved. With such precise methods, it is also possible to plan and incorporate vascular networks, hair follicles and sweat glands into the developed constructs to enhance tissue function and aesthetics after implantation (Jia et al. 2016).

Bioprinting allows the implementation of novel approaches in the treatment and patient care, for instance, surgeons may have control over cell/construct implantation at the micro- and millimetre scale with the help of automated robotic printers (Tran and Wen 2014). Unlike conventional scaffold fabrication technologies, 3D bioprinting allows the fabrication of custom made or personalized tissue constructs. This helps to deposit desired cell types with selected biomaterials and desired bioactive substances. Custom-made grafts are highly essential especially in skin reconstruction with complicated topography of organs like ears and breast (Li et al. 2016). Bioprinting allows the fabrication of structure with exact architecture, shape and amount to fit with defect to be treated (Richards et al. 2017).

The major steps in the bioprinting process are imaging of the tissue architecture to be constructed and design, selection of biomaterials and appropriate cells, and finally the printing of the tissue construct (Murphy and Atala 2014b). The printed construct will be kept under in vitro conditions for maturation, and then implanted to the intended site. Medical imaging technologies like computed tomography (CT) and magnetic resonance imaging (MRI) are important tools used by tissue engineers to gather information on 3D structure at the cellular, tissue or organ levels for bioprinting. Moreover, computer-aided design and computer-aided manufacturing (CAD–CAM) tools are also being used to generate complex 3D images for bioprinting. The basic biomaterial for bioprinting, the bioink is prepared in fluid form and then fed into the printer either in one mixture or separate portions that are mixed in the body or at the nozzle of the printer. One of the major challenges in the 3D bioprinting process is the selection of materials that provide the desired mechanical strength for tissue constructs while being biocompatible and printable. Materials currently used in the bioprinting are mainly based on natural polymers like alginate (Markstedt et al. 2015), gelatine (Bertassoni et al. 2014), collagen (Lee et al. 2010), fibrin (Pelaez et al. 2009) and hyaluronic acid (Pescosolido et al. 2011) or synthetic polymers like polylactic acid (Narayanan et al. 2016), or polyaprolactone (PCL) (Recek et al. 2016). Compared with natural polymers, a combination of synthetic polymers with various reinforcing agents, result in constructs with excellent mechanical properties (Gao et al. 2014).

Crosslinking of natural polymers are necessary to make them stable after bioprinting under physiological conditions (Carrow et al. 2015). Toughening of polymers after bioprinting can be achieved by using either physical or chemical crosslinking methods (Ozbolat 2016). Chemical crosslinking methods such as enzymatic (e.g., mushroom tyrosinase for gelatin) (Das et al. 2015), use of tannic acid (for collagen crosslinking) (Heijmen et al. 1997), divalent cations such as calcium ions (for alginate) (Tabriz et al. 2015) were widely used. Physical crosslinking methods such as ultraviolet treatment (e.g., for gelatine methacryloyl (GelMA) are also used for stabilizing the cell-laden bioprinted construct (Hassanzadeh et al. 2016).

The selection of appropriate cells for tissue or organ printing is critical for the success of the fabricated construct. Apart from the primary functional cells most tissues also contain cell types that provide assistive, barrier or mechanical functions to the tissue. For instance, pericytes are required to protect the endothelial cells in blood vessels (Caporali et al. 2017). Since multiple types of cells embedded within the same or different polymers need to be printed in parallel, many bioinks need to be prepared for each print. Since stem cells are totipotent, printing with stem cells will reduce the number of bioinks required for a particular bioprinting (Lei and Wang 2016). In order to avoid immune response, in clinical contests, cells would be isolated from the patients who need an implantation (Mandrycky et al. 2016). Such situations, stem cells isolated from the patient themselves with the inherent potential to proliferate and differentiate into any desired cell types are the most suitable and promising source of cells.

There are many instrumentation approaches in bioprinting such as microextrusion, Inkjet or laser-assisted bioprinting (Li et al. 2016). In microextrusion, biomaterial is extruded through bioprinter nozzles (Colosi et al. 2017). Microextrusion systems function by either pneumatic or mechanical (piston or screw) operational modules. In inkjet bioprinting, thermal, piezoelectric, or electromagnetic means are used for depositing small bioink droplets through the nozzles (Bishop et al. 2017). Inkjet bioprinter scan, achieve resolutions close to 50 μm but it lacks control over precise positioning of cells in bioprinted construct (Sears et al. 2016). Key advantage of inkjet bioprinting is the achievable speed and the major disadvantage is the requirement of liquid and less-viscous bioink (Hölzl et al. 2016). Recently, microfluidic systems were combined with extrusion printing for relatively easy deposition of multiple materials and resulted in high velocity printing (Hou et al. 2017). It is plausible that the extrusion-associated stress may affect cell viability (Kang et al. 2017). The major limitation of extrusion bioprinting is its low resolution (below 50 μm)(Ozbolat and Hospodiuk 2016). In laser-assisted bioprinting or biological laser printing (LAB), laser energy is used for volatizing sacrificial layer in the system to propel a payload to a receiving substrate (nozzle-free bioprinting) (Dababneh and Ozbolat 2014). LAB is characterized by excellent resolution and it has lower throughput, and it is slower than extrusion and inkjet printing modalities.

## Skin tissue biology, wound healing and regeneration

Skin is a complex heterogeneous organ with versatile structural and mechanical properties consisting mainly of the outermost epidermis and the underlying dermis (Kanitakis 2002). A subcutaneous adipose-storing hypodermis layer and various appendages such as hair follicles, sweat glands, sebaceous glands, nerves, lymphatics, and blood vessels are also present in the skin (Brohem et al. 2011). These multiple components of the skin ensure survival by carrying out critical functions such as protection, thermoregulation, excretion, absorption, metabolic functions, sensation, evaporation management, and aesthetics (Foda et al. 2011). Skin provides resistance to applied forces; however, it is also a dynamic material that can remodel its structure to respond to changes in the internal as well as external environment.

Microscopically, skin is a multilayered organ composed of many histological layers. It is generally subdivided into three layers; the epidermis, the dermis and the hypodermis. The uppermost nonviable layer of the epidermis, the stratum corneum, has been demonstrated to constitute the principal barrier to percutaneous penetration (Walker and Smith 1996). The excellent barrier properties of the stratum corneum can be ascribed to its unique structure and composition. The viable epidermis that lies beneath is responsible for the generation of the stratum corneum. Dermis lies exactly adjacent to the epidermis and is composed of a matrix of connective tissue, which endows the skin with its elasticity and resistance to deformation. The blood vessels that are present in the dermis nourish the skin with nutrients and oxygen (Michael et al. 2013). The hypodermis or subcutaneous fat tissue supports the dermis and epidermis and provides thermal isolation and mechanical protection to the body.

Several biomaterials have been used clinically to manage skin wounds. The traditional forms of wound dressings are non-resorbable gauze and/or sponge, which was then replaced by the advanced materials which comprise of thin films made of polyurethane that are permeable to vapour and gases (Augustine et al. 2014b). Many attempts have been made by the researchers to promote the regeneration of the skin using advanced concepts in bioengineering. There are many success stories on the skin regeneration with the aid of xenografts, allografts or autografts (Debels et al. 2015). Research in this field has brought novel biosynthetic materials and tissue-engineered living skin replacements which are being widely recognized as ‘skin substitutes’. However, the field is in its infancy to design and develop a fully functional multi-layered ‘artificial skin’ with all the layers of natural skin along with other appendages like blood vessels, sweat glands, sebaceous glands and hair follicles. Constructing a dermo-epidermal substitute that rapidly vascularizes, optimally supports a stratifying epidermal graft on a biodegradable matrix, and that can be conveniently handled by the surgeon, is now the ambitious goal (Braziulis et al. 2012). After all, this goal has to be reached coping with strict safety requirements and the harsh rules of the economic market. Therefore, the development of rationally designed fully functional skin substitute can have important implications, not in clinics, but also as an in vitro model for pharmaceutical and cosmetic testing. Several types of human skin recombinants, also called artificial skin that provide this critical 3D structure have now been reconstructed in vitro.

However, cell biologists, biochemists, bioengineers, and surgeons are still searching for novel approaches and tools for the generation of complex skin substitutes that can readily be implanted in large quantities, possibly in only one surgical intervention and without significant scarring (Kamel et al. 2013).

## Bioprinting of skin

Most tissue-engineered skins are created by expanding normal skin cells in the laboratory on porous biodegradable scaffolds (MacNeil 2007). Such engineered skin can be used for long time healing against the synthetic materials that can only be used for short time healing, because the materials must eventually be removed or to be replaced by natural skin cells (Powell et al. 2008). An ideal bioprinted skin should have certain attributes such as being biocompatible, desired mechanical properties to match the tissue, an appropriate surface chemistry and be highly porous with a network of interconnected pores that will allow cells to attach and be able to transport nutrients and remove wound exudates (Murphy and Atala 2014a).

**Fig. 1:**
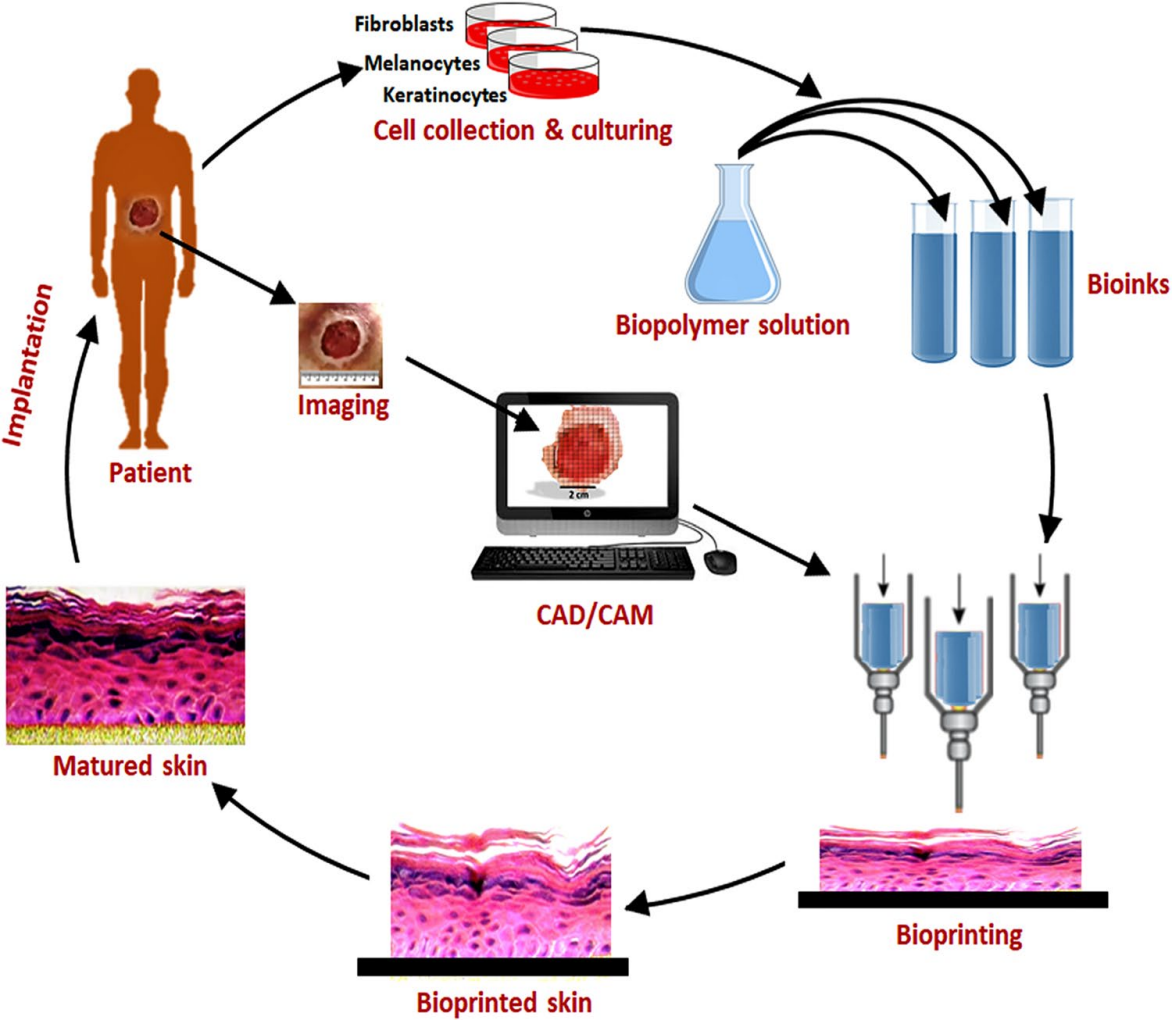
Steps in the fabrication of bioprinted skin. Various cells such as keratinocytes, fibroblasts and melanocytes would be collected from the patient and grow and multiply in cell culture system. A suitable biopolymer is mixed with the cells and the formed bioink is fed to the bioprinting system. Features of the wound are captured and a 3D structure is reconstructed using CAD/CAM approaches. According to the 3D pattern, wound tissue will be reconstructed, allowed for maturation in vitro and implanted back to the patient

A generalized schematic representation of various steps involved in skin bioprinting is shown in Fig. 1. When coming to the first step of skin bioprinting, the imaging of tissue to be reconstructed, unlike other organs like bone or breast, highly advanced techniques like CT scanning or MRI scanning may not be necessary. Most of the skin wounds are peripheral and directly visible to the tissue engineer and hence digital photographs or thermal images will be enough (Liu et al. 2016). Unless, if the skin substitute is intended for specific areas of the body such as ears, nipples, etc., a flat surface with a square, rectangular or circular shape can be fabricated and trimmed for specific implantation sites. 3D architecture of the skin to be reconstructed will be designed using appropriate CAD/CAM programs or specific 3D printing software (Tran and Wen 2014). Cells like keratinocytes, fibroblasts and melanocytes can be isolated from patients on body by a small biopsy. As in conventional tissue engineering, after in vitro culturing to achieve desired cell density, they will be mixed with a suitable biopolymer (e.g., alginate) and printed in a bioprinter. Alternatively, stem cells (e.g., mesenchymal stem cells (MSCs)) can also be collected from the patient and differentiated into various skin layers after printing. The printed skin construct will be allowed for the maturation under in vitro conditions which will then be implanted into the defected area of the patient.

## Desirable properties of bioprinted skin

Appropriate cell types and suitable biomaterials are the two major requirements to produce clinically viable bioprinted skin tissue. A shortcoming of most tissue engineered skin constructs is that they rely upon molecular diffusion and mechanical perfusion for nutrient supply. Since diffusion is generally limited to 100–200 μm, cell viability in the construct will be compromised (Tran and Wen 2014). Presence of a highly developed vasculature in the bioprinted construct can provide nutrients at the vicinity of the cells. In this scenario, development of vascularized cell-laden bioprinted skin substitutes would have great benefit in repairing skin defects (Bertassoni et al. 2014).

An ideal skin substitute should have an appropriate surface chemistry and should be highly porous with a network of interconnected pores that will allow cells to attach and be able to transport nutrients and remove wound exudates (Zein et al. 2002). A scaffold for tissue engineering with a large surface area to volume ratio will have higher opportunities for the cells to attach and migrate (Hutmacher et al. 2004). The porous structure of constructs will provide good aeration for the cells and does not lead to wound dehydration. At the same time, the pores need to be small so that the skin substitute will protect the wound from microbial invasion (Augustine et al. 2017b).

Characterization of physical properties such as porosity, mechanical strength and degradation rate are important to determine the suitability of bioprinted construct for tissue engineering applications. Ideal construct for engineered skin tissue would have high porosity with pore size of 200–400 μm that promotes tissue in growth in vivo (Park et al. 2016). Polyelectrolyte gelatin-chitosan hydrogel skin construct that has similar mechanical properties with skin tissue with high porosity showed good biocompatibility with fibroblast cells (Ng et al. 2016b).

Bioprinted skin constructs should be biodegradable but stable until the skin regeneration process is completed. They should be able to maintain its three-dimensional structure for at least 3 weeks to allow ingrowths of blood vessels, fibroblast and for epithelial cell proliferation (Sekine et al. 2013). Biodegradation should preferably take place after this period (Augustine et al. 2015). Moreover, degradation products should not create a massive foreign body response. Understanding and controlling the biodegradation is an important aspect for maintaining 3D architecture of any tissue engineering scaffold throughout the implantation period and subsequent integration with native host tissue (Augustine et al. 2016c).

Crosslinking mechanism of the polymer used for bioprinting is also a very important factor which affects the mechanical stability and degradation rate of the construct. Various hydrogels based on alginate, gelatine, collagen, chitosan and agarose have been used as a bioink for printing skin substitutes because of simplicity of their crosslinking mechanisms (Murphy et al. 2013). Alginate is a popular biologically derived and relatively inert bioink, which is generally crosslinked with calcium ions after bioprinting process and it degrades slowly within weeks to months depending on the degree of crosslinking (Jia et al. 2014). For instance, alginate preserves its strength and structure up to 3 weeks and is suitable for skin reconstruction (Sun and Tan 2013). On the other hand, 40 mM BaCl_2_ treatment was able to keep the structure in place over 7 days without the appearance of visible cracks within the grid structure (Tabriz et al. 2015). BaCl_2_ acted as a tertiary cross-linking agent and further improved the stability of the structure over 7 days.

Moreover, other general prerequisites for any tissue engineering scaffolds such as cell adhesion, cell proliferation and biocompatibility in all respects are also very important in skin bioprinting.

## Biomaterials in skin bioprinting

The biomaterial used for skin bioprinting should be printable, degradable, possess enough mechanical properties and biocompatible with immobilized cells (Müller et al. 2015). Most importantly, bioink needs to exist in two different phases and should be capable to change from one form to another. It should have a liquid phase with subsequent solidification to keep rigid form once printed. The solidification process of bioink should be slow enough to avoid clogging of the nozzle. However, if it sets very slowly the structure of the resulting construct will be affected (Xu et al. 2014). It should have adequate structural stability and strength as well as enough stability in aqueous media. Such bioink material should support and preferably enhance cell viability, distribution/migration, proliferation, differentiation and formation of appropriate tissue. It should allow cell–cell adhesion and paracrine signalling (Metcalfe and Ferguson 2007a). The biomaterial itself should be biocompatible and should also enhance cell attachment and migration. In addition, they should also be suitable for the incorporation of other materials and active agents that provide functional or structural support to the printed construct.

An appropriate bioink should have a storage modulus between 10^2^ and 10^3^ Pa in order to achieve effective printing (McBeth et al. 2017). Resulting constructs should have and able to maintain certain physical, chemical and biological properties such as adequate mechanical stability and structural rigidity to support the proliferation various cells. Mechanical properties of the construct should match with the skin tissue to be repaired (Ozbolat and Yu 2013). It should have also appropriate pore size, interconnected channels and pores for cell migration and fluid transport (Koch et al. 2015).

Though, various biomaterials can be printed including polymers, processing methods should be cytocompatible. As an example, although polymers like polylactic acid (PLA) and polycaprolactone (PCL) (Augustine et al. 2015) were widely used in tissue engineering, because of their relatively high processing (melting) temperature, such biopolymers would not be suitable for cell encapsulation and bioprinting. Hydrogels are used in bioprinting because of their low temperature gelation properties (Dai et al. 2017). They may be either natural such as alginate, chitosan, hyaluronic acid, fibrin, gelatin, etc. (Augustine et al. 2013) or synthetic such as Poloxamer 407 (Pluronic F-127)(Müller et al. 2013) or a combination of polyethylene glycol diacrylate and gelatine methacrylate (GelMA)(Wang et al. 2015). Various modifications and synthetic strategies like functionalization were used to tune the properties bioink to make them suitable for skin reconstruction.

There are many natural biomacromolecules used in tissue engineering applications (Augustine et al. 2013). Various properties of natural biomaterials are advantageous in skin reconstruction. As an example, collagen is characterized by possessing RGD sequence motifs which are important for keratinocyte attachment and wound healing (Rho et al. 2006). However, it does not preserve its original shape, has low mechanical properties and it suffers from batch-to-batch variations (Antoine et al. 2014). Thus, gelatin, a hydrolyzed form collagen, was used with hyaluronan (Skardal et al. 2010) or with chitosan (Ng et al. 2016b). Silk fibroin was blended with gelatin because silk fibroin has robust mechanical properties and tunable degradability while gelatine offers RGD sequences for cell adhesion and migration (Das et al. 2015).

Polyuronate derivatives like alginate and pectin find a robust position in biomaterial applications (Augustine et al. 2015). They are commonly used for bioprinting because of their cost effectiveness, biocompatibility, suitable viscosity and fast gelation rate. They form gel almost instantly through sodium–calcium ion exchange reaction which occurs at room temperature (Augustine et al. 2016a). Unlike collagen or gelatin, alginate lacks RGD motifs and it may need functionalization to enhance cell attachment and function (Plouffe et al. 2009). In a specific study using the blends of bioprinted alginate and gelatin in mouse full thickness wound model, demonstrated the efficacy to substantially enhance the rate of wound healing (Liu et al. 2016).

Studies show that even human blood plasma can successfully be used as bioink for the development of bilayered skin. Bioinks containing human plasma as well as primary human fibroblasts and keratinocytes that were obtained from skin biopsies were used for the fabrication of the skin substitute (Cubo et al. 2016). After implantation in mouse, it exhibited a characteristic wrinkled, thick and whitish tone, very similar to the appearance of native human skin and clearly different from the surrounding mouse skin. The fabricated skin was structurally also very similar to human skin. All the strata characteristic of normal skin, stratum basale, stratum spinosum, stratum granulosum and a well-developed stratum corneum were easily identified in the printed skin.

Polymer blending and making composites are of great interest in skin tissue engineering, since these approaches could lead to the development of a new range of biomaterials with desired properties to match with the that of native skin (Armentano et al. 2010). As a strategy to overcome the limitations associated with purely polymeric systems (e.g., inferior mechanical strength and lack of cell adhesion), nanocomposites have been introduced as possible alternatives to improve such limiting characteristics (Carrow et al. 2015). Mechanical properties of today’s available porous scaffolds are insufficient in terms of elastic stiffness and compressive strength compared to the human skin (Rezwan et al. 2006). Thus the strategies such as blending and making composites has been tried (Schuurman et al. 2011). Nanomaterials used along with polymers provide additional sites for cross-linking and stress distribution (Nandagopal et al. 2016) to improve mechanical stability (Moreno et al. 2010). Nanocomposites also provide appropriate stimulus for cell differentiation and proliferation (Tautzenberger et al. 2010). The polyelectrolyte gelatin-chitosan hydrogels formulated in this work was optimized for 3D bioprinting at room temperature to achieve high shape fidelity of the printed 3D constructs and good biocompatibility with fibroblast skin cells. Blending can also be used to enhance the functional property of the bioink. For instance, blending of alginate and gelatin enhanced the rate of wound healing in mouse full thickness wound model (Liu et al. 2016).

## Cells used in skin bioprinting

The gold standard cell source in skin bioprinting is autologous cells derived from the patient, which is then proliferated in the laboratory to obtain the desired cell numbers. On the other hand, there are different types of cells that can be used for skin bioprinting which can be cell lines, primary cells, or stem cells (pluripotent or multipotent). Pluripotent cells such as induced pluripotent stem cells (iPSCs) are attractive candidates since they are very versatile in addition to the ethical acceptance, they are genetically tailored to a patient (Smith et al. 2016). Recently, MSCs were also derived from iPSCs and represent attractive source because they can circumvent the limitations of conventional autologous MSCs obtained from bone marrow. iPSC-derived MSCs (iMSCs) are also rejuvenated during the reprogramming process with better survival, proliferation and differentiations capabilities (Liu et al. 2013). These advances in stem cell technologies may contribute to provide suitable cell source alternatives for the use in skin bioprinting. Studies show that iPSCs can be differentiated into various types of skin cells with the capacity to form multi-differentiated epidermis with hair follicles and sebaceous glands (Aasen et al. 2008).

In bioprinting, cells can be used as individually-encapsulated single cells, dispersed cells in the matrix gel or gel precursor or in microcarriers, cell aggregates (spheroids) (Colosi et al. 2017). Commonly used cells for skin bioengineering include mesenchymal stem cells and endothelial or endothelial progenitor cells (Augustine et al. 2017a). Since angiogenesis is an important factor that determines the success of skin tissue engineering, endothelial cells (ECs) were used along with other cells during printing. Although bone marrow derived stem cells are widely used for skin bioengineering, human inferior nasal turbinate tissue-derived mesenchymal stromal cells (hTMSC) cells were also used in bioprinting because of the advantages that they have (Das et al. 2015). Such cells have very high yield (~ 30 times more than adipose tissue derived MSCs at early passage (Shafiee et al. 2011) and high proliferation rate, five times higher than bone marrow derived stem cells (Bonab et al. 2006).

**Fig. 2:**
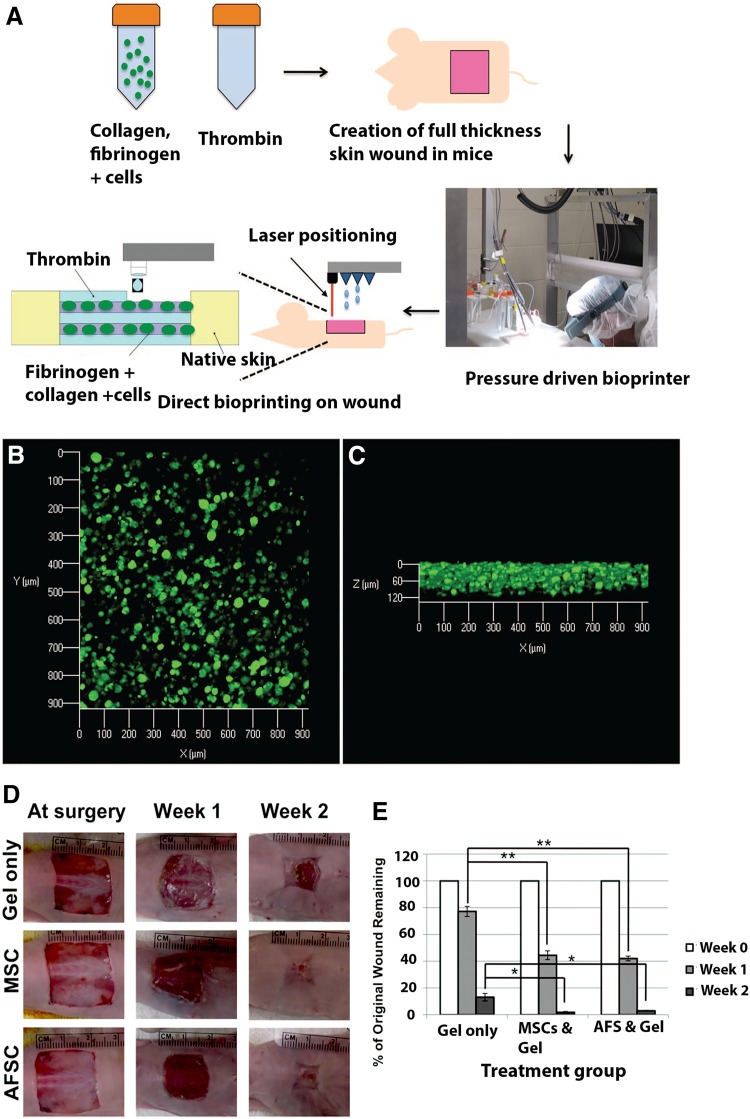
Bioprinting of stem cells for the treatment of skin wounds. **a:** A schematic describes the approach by which amniotic fluid-derived stem cells (AFSC) are bioprinted in order to increase healing of a full-thickness skin wound. Wounds containing the deposited gels with green fluorescent protein-tagged AFSC were harvested after 24 h of post-printing and analyzed with confocal microscopy. Images revealed evenly distributed cells in the gels, as viewed from the top **b** or from the side **(c)**. **d:** Gross histology images illustrating wound closure in gel-only, MSC, and AFS treatments. **e:** Percentage of unhealed wound remaining at the day of surgery, after one and 2 weeks. Abbreviations: *AFS* amniotic fluid-derived stem cells, *AFSC* amniotic fluid-derived stem cell, *MSC* mesenchymal stem cell. Adopted with permission from (Skardal et al. 2012)

High proliferative capacity and multilineage differentiation potential of amniotic fluid derived stem cells (AFS) was exploited for skin bioprinting (Fig. 2). AFS are immunocompetent cells and hence used for the direct bioprinting on skin wounds in mice (Skardal et al. 2012). Co-printing of AFS cells and bone marrow-derived mesenchymal stem cells (MSCs) in fibrin-collagen gel over the wound site leads to significantly higher wound closure and re-epithelialization. Histological examination showed increased microvessel density and capillary diameters in the AFS cell-treated wounds.

Das et al. (2015) (Das et al. 2015) used a blend of silk fibroin and gelatin combined with human inferior nasal turbinate tissue-derived mesenchymal stromal (hTMSC) cells for the bioprinting (extrusion). They observed a higher cell viability and multilineage differentiation of the encapsulated hTMSC in the scaffolds.

**Fig. 3:**
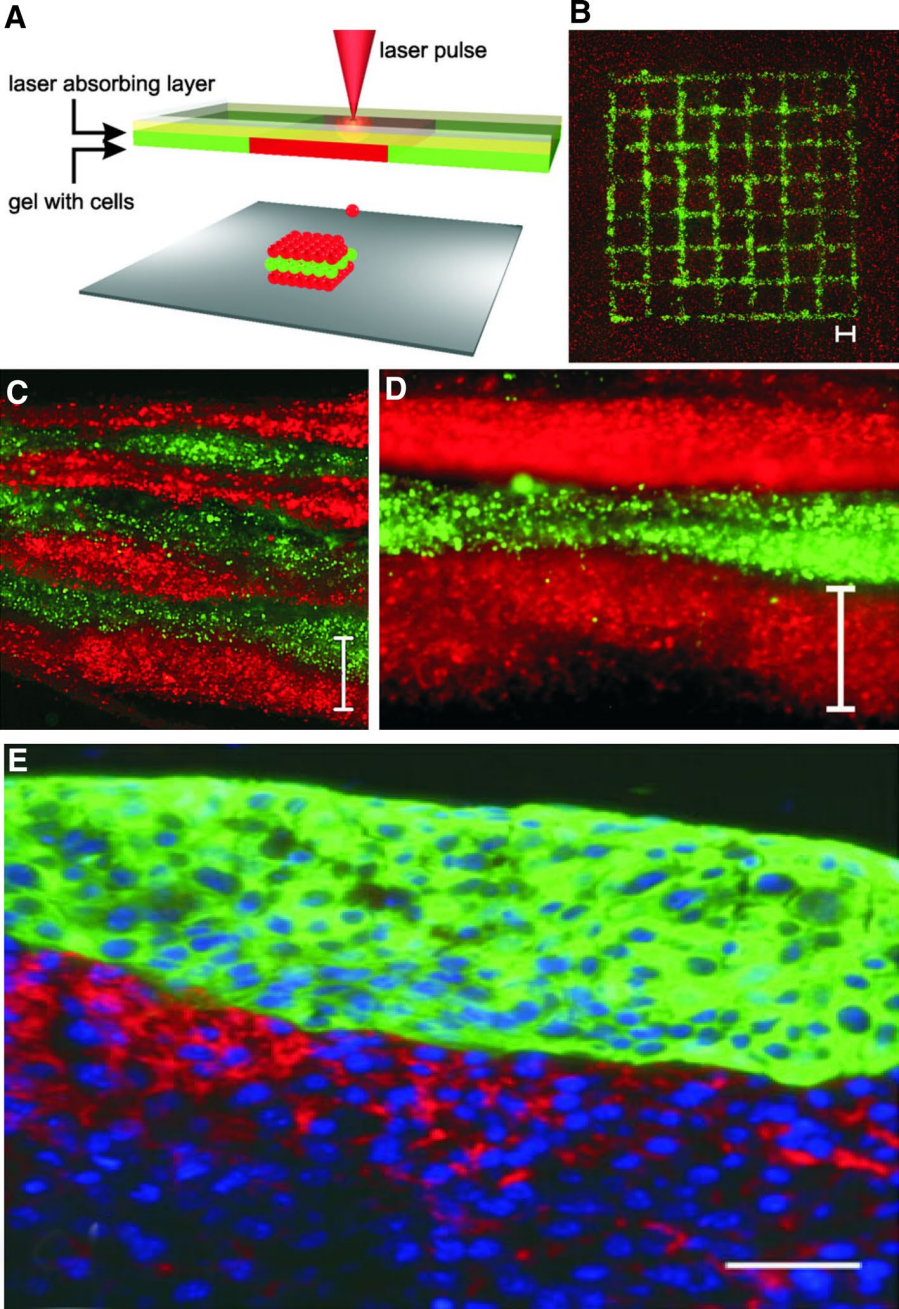
Sketch of the laser printing setup **a** A printed grid structure **b** of fibroblasts (green) and keratinocytes (red) demonstrates micropatterning capabilities of the laser printing technique. Seven alternating colour layers of red and green keratinocytes **c** and the magnified view **d**. Each colour layer consists of four printed sublayers. A histological section was prepared 18 h after printing. Scale bars are 500 µm. In picture **e** the fibroblasts are stained in red (pan-reticular fibroblast), keratinocytes are stained in green (cytokeratin 14) and cell nuclei are stained in blue (Hoechst 33342). In this case, scale bar is 50 µm. Adopted with permission from (Koch et al. 2012)

Cell viability is an important aspect for assessing the efficiency of bioprinting process and achieving tissue functionality. It is dependent on many factors such as bioprinting process, crosslinking method, cell source, bioink viscosity, porosity etc. Although microextrusion bioprinting is the most affordable and common technology, it provides the lowest cell survival rate of about 50% compared to that of inkjet- and laser-based bioprinting due to the extrusion associated pressure and shear stress (Murphy and Atala 2014a). Despite the use of time consuming and high cost printing system, the laser-based printing machines performs the highest cell survival and cell functions after printing. Recent studies show that thermal inkjet (Duarte Campos et al. 2016) and pressure extrusion (O’Connell et al. 2016) printing systems can provide more than 95% of cell viability after 3 weeks of post printing. Some crosslinking methods require toxic agents or conditions that may affect cells, which results in low cell viability and functionality. Generally, high viscous materials provide structural support for printed construct and lower-viscosity materials providing suitable environment for maintaining cell viability and function. Moreover, the choice of cell types is important for the proper functioning of bioprinted construct and mimicking native tissue. hMSC survival was > 98% in thermo-responsive collagen type I-agarose blend hydrogels fabricated using inkjet-based printing (Duarte Campos et al. 2016). Laser-assisted bioprinting (LaBP) was used to fabricate a fully cellularized skin substitute (Koch et al. 2012). In this approach vital cells were arranged in a 3D fashion by LaBP as multicellular skin graft analogue. For this purpose, fibroblasts and keratinocytes embedded in collagen were printed in 3D and evaluated different characteristics, such as cell localization and proliferation. Briefly, the experimental setup was consisted of two coplanar glass slides (Fig. 3 **(A)**). The top slide was covered underneath with a laser absorbing layer made up of a thin gold layer and a layer of cells embedded in collagen gel or a mixture of blood plasma and alginate. Laser pulses were focused through the glass slide into the absorption layer which was evaporated locally. The cell–hydrogel compound was propelled forward as a jet by the pressure of a laser-induced vapour bubble. Layer-by-layer a 3D cell pattern was generated. A Matriderm^®^ sheet was positioned on the lower glass slide to print cells on it. The advantage of this approach was that a multi-layered skin equivalent can be generated by the layer by layer deposition of fibroblasts and keratinocytes (Fig. 3c d e). Interestingly, the study demonstrated that the cells were adhered to each other by the successful formation of gap junctions. In a relatively similar study, researchers positioned fibroblasts and keratinocytes on the top of a Matriderm^®^ based stabilizing matrix (Michael et al. 2013). These skin constructs were subsequently tested in vivo, employing the dorsal skin fold chamber in nude mice. The transplants were placed into full-thickness skin wounds and were fully connected to the surrounding tissue when explanted after 11 days. The printed keratinocytes formed a multi-layered epidermis with beginning differentiation and developed stratum corneum. Proliferation of the keratinocytes was mainly detected in the suprabasal layers. These findings suggest that LaBP is an excellent bioprinting approach for the generation of bioprinted skin 3D constructs.

Zhang et al. investigated laser bioprinting-induced cell injury in NIH 3T3 mouse fibroblasts (Zhang et al. 2017). They found that minimum time required for cells to reach late apoptotic stage is 4–5 h after printing. Another interesting study investigated the effects of alginate gelation, gelation time, alginate concentration, and laser fluence on the post-transfer cell viability of NIH 3T3 fibroblasts (Gudapati et al. 2014). It was observed that the effects of gelation mainly depend on the duration of gelation. Two-min gelation was observed to maintain higher cell viability after 24 h incubation; however, 10 min gelation decreased the cell viability due to the formation of a thick gel membrane that hindered nutrient and oxygen diffusion from the culture medium. They also found that higher laser fluence or alginate concentration affects cell viability.

## Role of microenvironment in skin regeneration

Cell microenvironment, both at pre-printing and post-printing stages have crucial role in the ultimate success of the bioprinted skin construct. Various physicochemical elements play important role in the generation and maintenance of a suitable microenvironment in bioprinted construct. The importance of microenvironmental factors was recognized by previous studies and dynamic culture was used for mimicking the natural niche in native skin to produce structures like sweat glands (Huang et al. 2016). To achieve specific cell differentiation, they incorporated mouse plantar dermis and epidermal growth factor into gelatin and sodium alginate based 3D-ECM. Using recent advancements in microfluidics, various gradients of biomolecular cues can also be incorporated during printing.

In the initial stages of cell proliferation and differentiation, growth factors may be more crucial, however at later stages other factors like mechanical stimuli are also important. It has been demonstrated that MSCs have extreme sensitivity to tensile property of the scaffolds and suggested that soft matrices may be associated with neurological differentiation, while stiffer ones with myogenic and rigid ones with osteogenic differentiation (Engler et al. 2006). Constructs that mimic the natural tissue in mechanical properties may provide better cell bioactivity.

Manipulation of micro- and macroenvironment by electromagnetic stimulation is another important approach which may provide great opportunities in skin bioprinting. Role of electromagnetic activity of cells are well studied and reported (Cifra et al. 2011). There are even instances were skin regeneration was achieved using pulsed electrical stimulation (Hinsenkamp et al. 1997). More advanced differentiation of keratinocytes, evident from epidermal histology was observed in cultures exposed to electrical current. However, in contrast, they observed a higher keratinocyte migration and proliferation in control samples. These understandings may help scientists to develop electrically active and clinically viable bioprinted skin constructs.

The pyroelectric and piezoelectric nature of the epidermal layer of skin may help to find novel ways to manipulate skin tissue adaptation and remodeling (Athenstaedt et al. 1982). Guided cell movement and migration can be achieved by applying small electric fields, and consequently can improve in vivo skin healing (Cinar et al. 2009). Such studies demonstrated that exposure of wounds with0.9 kV/m to 1.9 kV/m chopped direct current (DC) electric field with a 30 micros repetition time favourably affected collagen synthesis and subsequent wound recovery. Gold nanorods were incorporated into GelMA hydrogel to render it conductive, showing improved cell adhesion and organization and cell-to-cell coupling (Zhu et al. 2017). Such approaches can be adapted with some modifications to use in skin bioprinting to improve cell adhesion and subsequent wound healing under in vivo conditions.

## Tissue integration and remodelling and angiogenesis in bioprinted skin constructs

A challenging issue that hinders the skin regeneration is the lack of blood vessel formation, which makes it difficult to develop bioprinted constructs that can biologically fulfill the requirements for skin regeneration (Xiong et al. 2017). Formation of robust, highly branched, interconnected capillaries that mimic in vivo vasculature is a crucial factor for the successful development of fully functional engineered skin construct (Augustine et al. 2017c). To develop functional skin tissue using 3D bioprinting, it is necessary to allow not only the formation of vascularized constructs but also the networking of them by anastomosis with host vasculature. Towards this goal, Chen et al. achieved to generate dense microvasculature in 3D hydrogels by encapsulating ECs and hMSCs in gelatin hydrogel (Chen et al. 2012). A recent study also explored the fabrication of complex vascularized skin constructs, comprised of a hard structure surrounded by a soft organic matrix (Cui et al. 2016). Bioprinting was performed using a dual 3D bioprinting platform consisted of a FDM 3D bioprinter and a SLA 3D bioprinter, using alternate deposition of biodegradable polylactide (PLA) fibers and cell-laden gelatin methacrylate (GelMA) hydrogels. Bioactive factors such as BMP2 and VEGF were incorporated into the construct to simultaneously promote cell proliferation and angiogenesis through the construct. In skin bioprinting, to improve angiogenesis, a gelatin-sulfonated silk composite scaffold that was incorporated with basic fibroblast growth factor 2 (FGF-2) was developed and tested (Xiong et al. 2017). Along with enhanced granulation tissue formation and the regeneration of skin-like tissues after implantation in rat skin defects, these scaffolds also stimulated dermal vascularization. Their findings thus demonstrate that addition of FGF-2 into the 3D printed constructs is a highly promising approach for enhancing skin regeneration.

## Challenges in skin bioprinting

Although there is a huge progress and potential which is evident from the recent advances in skin bioprinting, several barriers still remain which limit the clinical translation of the engineered construct. The most critical challenge is the need of large skin construct with highly developed vasculature (Hendrickx et al. 2011). Blood vessel anastomosis is critical for the long term potency of the construct after implantation. Reducing the period between the post implantation early stage and the angiogenesis is a critical challenge to be addressed. This is a decisive period that will determine the success of any graft which is implanted in the body. Further, the requirement of a bioprinted skin with multi-layered complex structure of the intact skin is still remaining as a big challenge (Groeber et al. 2011). It is most important to maintain the thickness and texture of epidermal, dermal and hypodermal layers of the bioprinted skin in such a way to match with the native skin of the patient. Skin constructs containing multiple functional structures such as sweat glands, hair follicles, sebaceous glands are still a difficult to achieve yet very important requirement. In future, it is most important to engineer fully functional skin by bioprinting such structures in a manner biomimicking native anatomy and physiology of skin and surrounding tissues. Maintenance of optimum mechanical properties, adequate resorbability of biomaterials in the construct, substitution by remodelling, population by living cells, integration with host tissue and long-term patency of the substitute are also very important.

Controlled and smart release of active molecule in spatial and temporal fashion is very critical for the successful regeneration of the skin defect. Recent advancements may come up with combination of solutions such as the use of strong fixation devices along with the engineered construct, use of reinforcement strategies with slow degrading or even partially degrading materials, vascularized flaps, incorporation of nanoparticulate angiogenic agents (Augustine et al. 2014a, 2017a) etc. can be adopted to overcome the present challenges in skin bioprinting. To facilitate translation of skin bioprinting to the clinic, ongoing and future developments should address these challenges and focus on defined and specific clinical applications. Moreover, regulatory bodies require precisely defined manufacturing processes and protocols before going to the clinical translation. We hope a multidisciplinary approach where mechanical engineers, bioengineers, material scientists, biologists, medical practitioners (such as plastic and reconstructive surgeons) and regulatory bodies will join hands to hand and work towards the development of 3D bioprinted constructs which will solve all the barriers on the way.

## Future perspectives of skin bioprinting

Skin bioprinting has the great opportunities to build complex tissue constructs needed to rectify complicated skin defects which are difficult to heal by normal clinical procedures. The final goal of bioprinting technology is the construction of a fully functional skin equivalent with vascular channels and all necessary appendages (hair follicles, sweat glands, sebaceous glands) by the simultaneous printing of cells and other agents, subsequently transplanted and anastomosed with native blood circulation. Unless otherwise we reached this goal, there are a lot of gap areas to fill to complete this mission. In order to facilitate the clinical translation of bioprinting, hand held device (Biopen) was developed for intraoperative bioprinting (O’Connell et al. 2016). In near future such instruments may find applications such as the precise deposition of cells in the wounds along with biopolymers in clinic. Another possible ground-breaking advancement will be 4D printing (Choi et al. 2015) which will gradually mature into 4D bioprinting (Gao et al. 2016; An et al. 2016). Stimuli responsive smart materials may also provide special properties such as shape memory even triggered shape memory to the bioprinted skin constructs.

Integration of biosensor technology with bioprinting may generate skin on a chip which may have immense potential in the study of pathophysiology of skin defects and drug screening for skin diseases (Ataç et al. 2013). Such systems can simulate inflammation, edema and test drug-based treatment (Wufuer et al. 2016). Recently, bioprinting has been used for the fabrication of organ-on-a-chip as it enables the printing of multiple materials, including biocompatible materials and even live cells in a programmable manner with a high spatial resolution (Yang et al. 2017). We are optimistic in the sense such innovation may happen in the near future in skin bioprinting which may further widen the possibilities and opportunities in studying the pathophysiology of skin related ailments and will aid in new drug discovery.

Despite the advancements in the use of differentiated cells (keratinocytes, fibroblasts and melanocytes) and multipotent stem cells (MSCs, BMSCs) in bioprinting of skin tissue constructs, there are great opportunities for induced pluripotent stem cells (iPSCs) and may find new possibilities to use them in skin bioprinting. Such researches are still at infancy and the outcomes are still awaited. Next will also be the combination of various cells that are usually found in the skin layers, and its related tissues, e.g. keratinocytes, epithelial cells, fibroblasts, endothelial cells, pericytes, neural cells, ligament cells, etc. Though much is needed to do to combine all of them in customized manner to produce intact native skin tissue constructs. Future research may also attempt to develop specific bioprinted constructs which may contain growth factors or anti-inflammatory drugs for diabetic wound healing.

Finally, combining state of art tissue engineering strategies and achievements made by current and ongoing research are highly promising towards the development of fully functional bioprinted skin, however, the field needs joint effort of several disciplines, convergence of different fields and the continued generous funding to see the successful translation to the clinic.

## Conclusion

Bioprinting is an additive biomanufacturing technology evolved from 3D printing that allows simultaneous printing of biomaterial matrix and cells to make functional tissue equivalents. The major steps in the bioprinting process are imaging of the tissue architecture to be constructed and design, selection of biomaterials and appropriate cells, and finally the printing of the tissue construct. Skin bioprinting utilizes cells, biomaterials and other active ingredients to produce viable constructs in a well-controlled fashion that successfully developed multilayered skin substitutes in vitro and in vivo. There are wide ranges of natural and synthetic polymers are used as matrix materials for skin bioprinting. Various cell types including keratinocytes, fibroblasts, MSCs, iPSCs can be used in the bioink as the source of cells. However, such constructs were limited by poor vascularization, lack of hair follicles and other appendages which are necessary for skin function and aesthetics. In order to overcome these shortcomings, cues such as mechanical/electrical stimuli, nanoparticles or growth factors are being used for mimicking the native skin natural niche in the construct. The structural complexity of bioprinted skin constructs and long in vitro, in vivo and clinical studies may delay regulatory approvals and a rational approach using already tested and approved material will help to simplify clinical translation. A collective effort combining various technologies and advances such as the use of conductive polymers, nanocomposites, 3D/4D bioprinting and microfluidics will help to accomplish the mission of fully functional bioprinted skin. This could be a promising approach to achieve structurally, functionally and aesthetically similar tissue to intact skin. Further, in addition to the need of technical and outcome standardization, rigorous randomized controlled trials and long term follow up data are required to determine the potency and oncological risk.
